# Comparison of Locations and Frequencies of Referred Pain in Pediatric Hip Disorders: A Retrospective Study

**DOI:** 10.7759/cureus.79214

**Published:** 2025-02-18

**Authors:** Tetsuhiro Hagino, Masanori Wako, Tetsuo Hagino, Jiro Ichikawa, Taro Fujimaki, Hirotaka Haro

**Affiliations:** 1 Department of Orthopaedic Surgery, National Hospital Organization (NHO) Kofu National Hospital, Kofu, JPN; 2 Department of Orthopaedic Surgery, University of Yamanashi, Chuo, JPN

**Keywords:** legg-calvé-perthes disease, pediatric hip disorders, referred pain, slipped capital femoral epiphysis, transient synovitis of the hip

## Abstract

Introduction: Children with hip joint disorders such as slipped capital femoral epiphysis (SCFE), Legg-Calvé-Perthes disease (LCPD), and transient synovitis of the hip (TSH) often experience pain in the hip, thigh, and knee. However, few studies have compared the specific locations and frequencies of pain associated with these disorders. Understanding these patterns can aid in diagnosis and management. This study aimed to fill this gap by comparing pain localization and frequency in pediatric hip disorders.

Methods: We retrospectively analyzed the medical records of 77 patients diagnosed with SCFE, LCPD, or TSH between 2011 and 2023. Pain location data were collected at the initial examination, and pain location in each disorder was compared.

Results: Age and sex distribution varied among diseases, but the referred pain location in each of the typical hip diseases in children, SCFE, LCPD, and TSH, and hip pain accounted for approximately 30-40%, knee pain was approximately 10-20%, and thigh pain was approximately 20-30% in each disease. The mean age of patients reporting hip and knee pain was similar, while those experiencing thigh pain tended to be slightly older. No significant differences in age were observed across different pain locations. Additionally, no significant sex differences were found in pain localization, with similar distributions in male and female patients. The distribution of pain locations among the three diseases showed no statistically significant differences, with hip pain being the most commonly reported site, followed by thigh pain and knee pain.

Conclusions: Despite the expected differences, pain localization in SCFE, LCPD, and TSH showed similar patterns, resembling adult hip disorders. This study highlights the consistent referred pain patterns across pediatric hip disorders, emphasizing their clinical significance in improving diagnostic accuracy.

## Introduction

Patients with hip joint disease sometimes present with pain around the hip joint, thigh, knee, and lower legs. These are considered to be referred pain; classically, hip osteoarthritis is also accompanied by groin pain and pain below the knee. In addition to hip pain, patients with hip osteoarthritis have been reported to experience thigh pain in 10-57%, knee pain in 2-35%, and buttock pain in 9-21% [1-3]. Although referred pain to the thigh or knee is a well-recognized phenomenon in hip joint pathology, there is a paucity of literature comparing the specific locations and frequencies of pain in different pediatric hip disorders.

Hip joint disorders in children, such as slipped capital femoral epiphysis (SCFE), Legg-Calvé-Perthes disease (LCPD), and transient synovitis of the hip (TSH), present unique challenges in diagnosis and management because of their diverse clinical manifestations. Among these challenges is variability in pain presentation, which often complicates accurate diagnosis and timely intervention. Millis reported that 35% of patients with SCFE present with thigh pain and 26% with knee pain [4]. Matava et al. and Kocher et al. also reported that patients presenting with pain other than hip pain were more likely to be diagnosed with delayed and severe pain [5,6]. Understanding the distinct patterns of pain localization in SCFE, LCPD, and TSH could provide valuable insights into their pathophysiologies and aid in clinical decision-making. Furthermore, this knowledge may facilitate earlier recognition of these conditions, leading to improved outcomes and reduced morbidity in affected children. Therefore, this study aimed to compare the localization and frequency of pain in pediatric hip disorders, shed light on their unique clinical profiles, and guide future diagnostic and therapeutic strategies.

## Materials and methods

Patients who visited the Department of Orthopaedic Surgery, University of Yamanashi Hospital, Chuo, Yamanashi Prefecture, Japan, between 2011 and 2023 and were diagnosed with SCFE, LCPD, or TSH were included in the study. We excluded cases with unclear descriptions of the pain location at the initial examination in the medical record. Patients under three years old were also excluded because of the uncertainty in confirming the pain location. Finally, 77 patients (18 with SCFE, 16 with LCPD, and 43 patients with TSH) were included in this study.

We investigated the location where the participants felt the most severe pain at the time of their first visit in their medical records and classified the pain location into the hip (including the groin, but not separately distinguishing buttock or lateral trochanteric pain), thigh, knee, and other locations. We also examined the proportion of boys and girls, average age, and distribution of pain locations for each disease.

As a statistical investigation, the χ2 test was used to test for differences in the distribution proportions of each survey item. The Yates correction was applied where necessary. The Kruskal-Wallis test was used to compare the age at the first visit between the groups. The statistical significance level was set at p<0.05.

This retrospective study was approved by the Institutional Review Board of the University of Yamanashi (approval number: 2797) and was conducted using the medical records of eligible cases at the University of Yamanashi Hospital, Chuo, Yamanashi Prefecture, Japan. The need for informed consent was waived by the Institutional Review Board owing to the retrospective observational design of the study.

## Results

Table 1 summarizes the age, sex, and disease status of all the patients. Due to the characteristics of the diseases, there was a distribution bias in age and sex for each condition, and the age at the first visit was significantly different among the disease groups.

**Table 1:** Patient characteristics SCFE: slipped capital femoral epiphysis; LCPD: Legg-Calvé-Perthes disease; TSH: transient synovitis of the hip

	SCFE	LCPD	TSH	Total
Male/female	14/4	15/1	33/10	62/15
Age (years, mean±SD)	11.9±1.7	7.2±1.9	7.5±2.7	8.4±3.0

**Figure 1:**
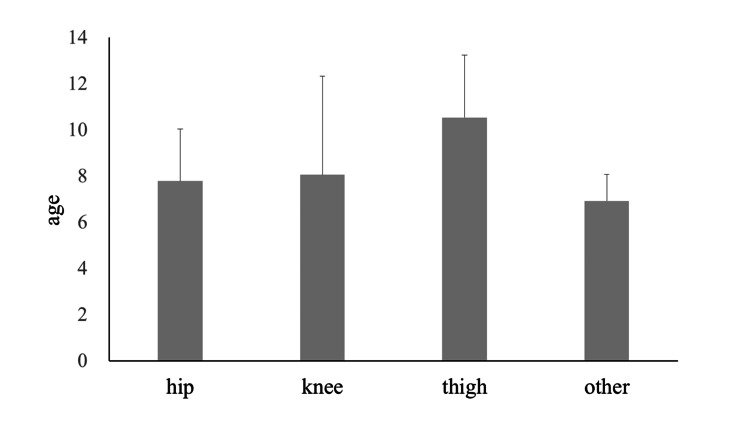
Age at initial examination by pain location The Kruskal-Wallis test was used. No significant differences were found between the groups (p=0.140).

Regarding the distribution of age by pain location, patients who reported hip and knee pain had similar mean ages, whereas those with thigh pain tended to be slightly older. However, statistical analysis revealed no significant differences in age among different pain locations (Figure 1).

**Figure 2:**
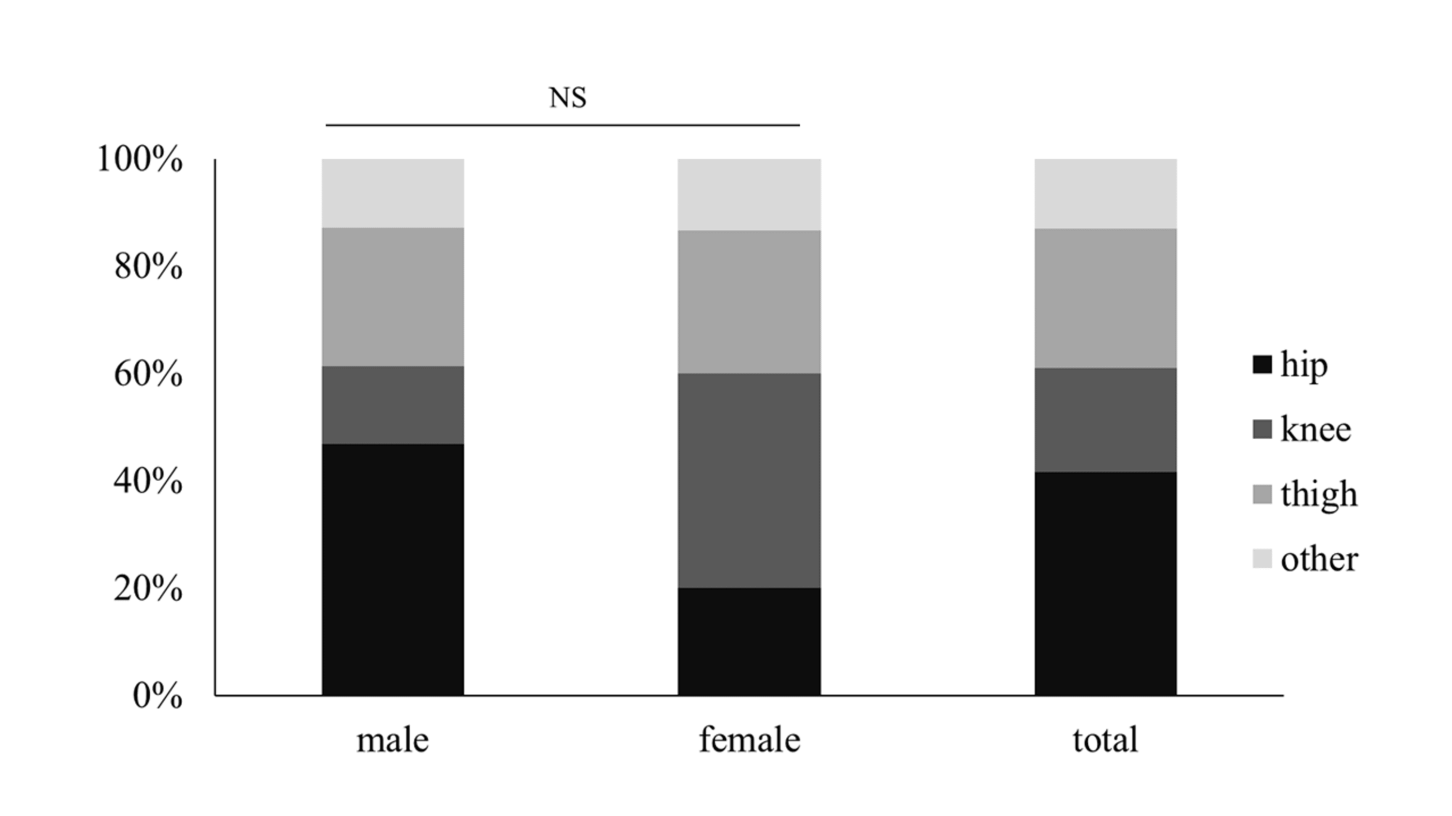
The distribution of pain locations by gender The χ2 test was used. There was no significant difference in the distribution of the pain locations between male and female patients (p=0.106). NS: not significant

In terms of pain location distribution by sex, no significant differences were observed between male and female patients. The proportions of hip, thigh, and knee pain were consistent regardless of sex (Figure 2). 

**Figure 3:**
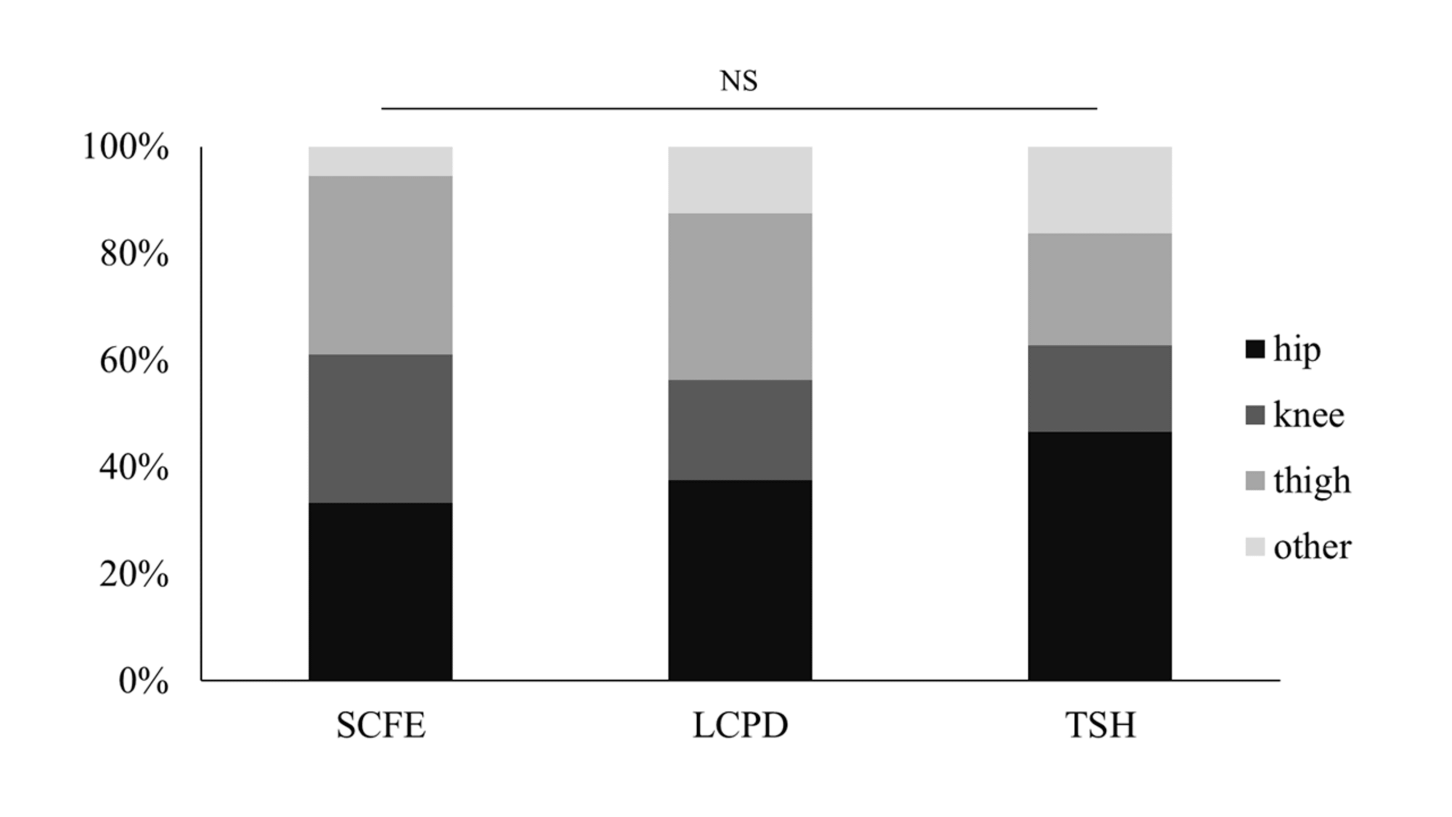
The distribution of diseases for each pain location and the distribution of pain locations for each disease The χ2 test with the Yates correction was used. No significant differences were found between the diseases (p=0.734). SCFE: slipped capital femoral epiphysis; LCPD: Legg-Calvé-Perthes disease; TSH: transient synovitis of the hip; NS: not significant

For the distribution of pain locations in each disease, although some variations were observed, no significant differences were found among SCFE, LCPD, and TSH. Hip pain was the most commonly reported site, followed by thigh pain and knee pain. The expected disease-specific differences in pain localization were not evident (Figure 3).

## Discussion

In this study, we investigated and compared the location of referred pain in each of the typical hip diseases in children, SCFE, LCPD, and TSH, and found that hip pain accounted for approximately 30-40%, knee pain for approximately 10-20%, and thigh pain for approximately 20-30% of each disease. The results showed no difference in pain location between the diseases. Referral hip joint pain in this study was defined as pain occurring at a site other than the hip joint at the time of diagnosis, which improved along with the resolution of the primary hip disease. Referral hip joint pain is thought to most commonly occur in the groin and anterior thigh. Referred pain in the thigh and knee seen in patients with hip disease, arm pain in patients with angina pectoris, and shoulder pain in patients with cholecystitis are thought to be caused by nociceptive dorsal horn neurons receiving convergent input from various tissues; thus, higher centers are unable to identify the true source of input [7]. The anterior hip joint is primarily innervated by the femoral and obturator nerves, while the posterior part is innervated by the sciatic nerve [8]. Additionally, a small percentage (1.6%) of dorsal root ganglion neurons project to both the hip and knee joints, which may contribute to referred pain between these two regions. Using double fluorescent labeling techniques, Miura et al. showed that fibers from the dorsal root ganglion are partially distributed around the hip and knee [9].

Some reports show the distribution of nerve endings in the hip joint capsule. Tomlinson et al. stated that increased proprioceptive and nociceptive functions may be present superior-laterally compared to other regions [10]. Moreover, Laumonerie et al. stated that the anterior capsule, primarily supplied by the femoral and obturator nerves, and superior labrum appear to be the primary pain generators of the hip joint, given their higher density of nociceptors and mechanoreceptors [11]. The anterior part of the hip capsule is innervated mainly by the femoral and obturator nerves, whereas the posterior part is innervated by the sciatic nerve [8,11].

All three diseases studied were pediatric hip diseases, but each had a different pathogenesis. In SCFE, a pathology similar to fractures associated with the disruption of the proximal femoral epiphysis is expected, and the anterior superior labral area is damaged due to femoroacetabular impingement associated with morphological changes in the femoral head [12,13]. The main pathogenesis of LCPD is thought to be crushing of the femoral head and synovitis [14,15]. TSH is associated with more severe inflammation and effusion of the hip joint than LCPD [16-18]. Thus, each disease is expected to have a different pathogenesis, damage location, and inflammation exposure. If each disease has a different site of injury within the hip joint, and if the innervation of the hip joint capsule differs from region to region, the distribution of referred pain would be different for each disease; however, the results of this study were different. Several previous surveys on adult patients with hip osteoarthritis cases have reported the location and frequency of referred pain. Lesher et al. [3] showed that patients with hip pathology awaiting fluoroscopically guided intra-articular hip injections most commonly presented with referred pain in the buttocks (71%), thighs (57%), groins (55%), or lower legs (16%).

Similarly, Hsieh et al. reported in a retrospective study of 113 patients who underwent total hip arthroplasty that thigh pain was present in 38%, buttock pain in 36.3%, and knee pain in 35.4% of patients [1]. Notably, the distribution and rates of referred pain in all three pediatric hip diseases examined in this study were not significantly different from those in adults. These results suggest that regardless of which part of the hip joint is damaged, inflammation of the hip joint and its chemical stimuli act on nerve endings in the joint capsule, which may result in hip, thigh, and knee pain at similar rates, regardless of hip disease. Further studies with more patients and disease groups may lead to a better understanding of pain mechanisms in the hip joint and possible ways to improve pain.

Furthermore, delayed diagnosis of several pediatric hip disorders can affect treatment outcomes. In SCFE, delayed diagnosis leads to progressive slippage and severe condition [5,6]. Early diagnosis and treatment of LCPD can help prevent bone deformity of the femoral head [19]. Septic hip arthritis, which can be difficult to distinguish from TSH, is also reported to be treated within five days of onset to prevent sequelae [20,21]. Although it is well known that an early and correct diagnosis is important in pediatric patients with hip arthritis, diagnosis is often delayed because of pain in areas other than the hip joint. As mentioned in the Introduction, this problem is particularly significant in SCFE, and the time required to establish a diagnosis is significantly longer in patients presenting with pain other than in the hip joint [22,23]. The results of this study, which showed that a certain percentage of children had referred pain in the thigh or knee, regardless of the type of hip joint disease, are useful findings that will aid in making an accurate diagnosis as early as possible.

This study has several limitations. First, the number of SCFE and LCPD cases was small. We would like to conduct further studies in the future with a larger number of cases to ascertain whether there are any differences in the results. Second, some cases involved more than one location of pain. However, by examining the most painful site, we were able to facilitate comparisons between our cases and previous reports. Third, because the pain location was based on self-reports from pediatric patients, precise differentiation of pain subregions (such as distinguishing between buttock pain and lateral trochanteric pain) was not feasible. Given the retrospective nature of this study and the challenges of obtaining detailed pain descriptions from children, this limitation should be considered when interpreting the results.

## Conclusions

We compared the pain locations in patients with SCFE, LCPD, and TSH, a pediatric hip disorder. Similar to previous reports on adults, some children had referred pain in the thigh or knee, regardless of the type of hip joint disease.
